# Коэкспрессия глутаматергических генов и генов аутистического спектра в гиппокампе у самцов мышей с нарушением социального поведения

**DOI:** 10.18699/VJ20.42-o

**Published:** 2020-03

**Authors:** И.Л. Коваленко, А.Г. Галямина, Д.А. Смагин, Н.Н. Кудрявцева

**Affiliations:** Федеральный исследовательский центр Институт цитологии и генетики Сибирского отделения Российской академии наук, Новосибирск, Россия; Федеральный исследовательский центр Институт цитологии и генетики Сибирского отделения Российской академии наук, Новосибирск, Россия; Федеральный исследовательский центр Институт цитологии и генетики Сибирского отделения Российской академии наук, Новосибирск, Россия; Федеральный исследовательский центр Институт цитологии и генетики Сибирского отделения Российской академии наук, Новосибирск, Россия

**Keywords:** RNA-seq, autism, hippocampus, glutamatergic genes, autism-related genes, social experience, RNA-seq, аутизм, гиппокамп, гены аутизма, глутаматергические гены, социальный опыт

## Abstract

В настоящее время существует представление о вовлеченности глутаматергической системы (ГГ) в механизмы развития аутизма. В предыдущих исследованиях нами было показано, что негативный социальный опыт, приобретенный в ежедневных межсамцовых конфронтациях, приводит к нарушениям в социальном поведении: снижению коммуникативности, нарушению социализации, появлению стереотипных форм поведения, которые могут рассматриваться как симптомы аутистического спектра. В связи с этим целью нашей работы было изучение с помощью транскриптомного анализа изменений экспрессии генов, кодирующих белки, вовлеченные в функционирование глутаматергической системы, и генов, связанных с патологией аутизма (ГА), в гиппокампе. В эксперименте использовали животных с нарушениями социального поведения, вызванными повторным опытом социальных побед или поражений в ежедневных агонистических взаимодействиях. Для формирования групп животных с контрастными типами поведения использовали модель сенсорного контакта (хронического социального стресса). Полученные образцы мозга были секвенированы в ЗАО «Геноаналитика» (http://genoanalytica.ru/, Россия, Москва). Транскриптомный анализ показал, что у агрессивных животных снижается экспрессия генов Shank3, Auts2, Ctnnd2, Nrxn2, для которых показано участие в развитии аутизма, а также глутаматергического гена Grm4. В то же время у животных с негативным социальным опытом экспрессия ГА Shank2, Nlgn2, Ptcdh10, Reln, Arx возрастает. При этом ГГ (Grik3, Grm2, Grm4, Slc17a7, Slc1a4, Slc25a22), за исключением гена Grin2a, повышают свою экспрессию. Корреляционный анализ выявил статистически значимую взаимосвязь из- мененной экспрессии ГГ и ГА. Полученные результаты, с одной стороны, могут служить подтверждением участия ГГ в патофизиологии развития симптомов аутистического спектра, с другой – свидетельствовать о коэкспрессии ГГ и ГА в гиппокампе, развивающейся под влиянием социальной среды. Так как большинство ГА, изменивших экспрессию в настоящем исследовании, являются генами, связанными с клеточным скелетом и внеклеточным матриксом, в частности участвующими в формировании синапсов, а ГГ, изменившие свою экспрессию, – генами, кодирующими субъединицы рецепторов, то можно предположить, что вовлечение ГГ в патофизиологию аутизма происходит на уровне рецепторов.

## Abstract (en)

There is a hypothesis of the involvement of the glutamatergic system in the development of autism. It has been shown that the chronic experience in daily intermale confrontations leads to disturbances in social behavior: a decrease in communicativeness, disturbances of socialization, emergence of stereotypical behaviors that can be considered as symptoms of the autistic spectrum disorders. So, the aim of this study was to investigate changes in the expression of glutamatergic (GG) and autism-related (GA) genes in the hippocampus of animals with impaired social behavior caused by repeated experience of social defeat or aggression in daily agonistic confrontations. To form groups of animals with contrasting behaviors, a model of sensory contact (chronic social stress) was used. The collected brain samples were sequenced at JSC Genoanalytica (http://genoanalytica.ru/ , Moscow, Russia). Transcriptomic analysis revealed a down-regulation of autism-related (Shank3, Auts2, Ctnnd2, Nrxn2) and glutamatergic (Grm4) genes in aggressive mice. At the same time, the expression of GA-related genes (Shank2, Nlgn2, Ptcdh10, Reln, Arx) and GG genes (Grik3, Grm2, Grm4, Slc17a7, Slc1a4, Slc25a22) excluding Grin2a was increased in defeated mice. Correlative analysis revealed a statistically significant association between GG and GA expression. These results can serve as a confirmation of the participation of the glutamatergic system in the pathophysiology of the autistic spectrum disorder

## Введение

Полагают, что выраженные нарушения в социальном поведении могут свидетельствовать об аутизме, который проявляется в детском возрасте и представляет собой нарушения развития нервной системы (Bauman, Kemper, 2005; Zablotsky et al., 2015). Согласно DSM-IV (American Psychiatric Association…, 1993), диагностические критерии аутизма включают триаду основных признаков: ухудшение социализации, под которой можно понимать способность индивида адекватно встраиваться в социум в новой обстановке, низкий уровень общительности и повторяющееся/стереотипное поведение. Хотя изучение близнецов свидетельствует о высокой наследуемости аутизма (Hallmayer et al., 2011), ни один ген не определен как единственная причина развития этого заболевания. Недавние геномные и генетические исследования показали, что сотни генетических вариантов, включающие общие и редкие взаимодействия генов, способствуют возникновению аутизма (Miles, 2011). Согласно различным базам данных (http://omim.org/, http://www.genecards.org/, http://autism.mindspec.org/autdb/Welcome.do http://www.malacards.org), в базе генов аутизма насчитывается около 1500 генов, которые в той или иной мере вовлечены в механизмы аутизма. Многие гены, связанные с развити- ем головного мозга, – потенциальные гены-кандидаты аутизма, к ним относятся гены и семейства генов Shank (1–3), Nlgn, Reln, Arx, Pcdh, Mecp2, Auts2 (Kleijer et al., 2014; Liu et al., 2015).

Литературные данные свидетельствуют об участии различных нейромедиаторных систем (например, серо- тонергической, дофаминергической) в развитии аутизма (Pavăl, 2017). В последнее время большое внимание уделяется глутаматергической гипотезе аутизма (Rojas, 2014). В пользу этой теории говорит то, что глутамат является одним из наиболее распространенных нейротрансмиттеров в мозге млекопитающих, его рецепторы сосредоточены в областях мозга (мозжечок, гиппокамп, префронтальная кора), в которых обнаружены нейропатологические изменения при аутизме. Во взрослом мозге глутаматные рецепторы участвуют в обучении и памяти (Riedel et al., 2003; Simonyi, 2010). Глутамат-опосредованные межнейронные взаимодействия также играют роль в формировании эмоционального поведения (Morgane et al., 2005; Faure et al., 2010). Показано, что для больных аутизмом характерен повышенный уровень глутамата в плазме крови, который может даже служить одним из биомаркеров этого заболевания (Zheng et al., 2016). У пациентов с аутизмом обнаруживаются и молекулярно-генетические повреждения глутаматергической системы (ГГ). Так, например, есть сообщения о мутациях в генах глутаматного рецептора GluR6 (Jamain et al., 2002), а также митохондриального переносчика глутамата (AGC1, ген Slc25a12) (Ramoz et al., 2004). Изменения наблюдаются на уровне м-РНК, белков- транспортеров и рецепторов ГГ в посмертных образцах мозга больных аутизмом (Purcell et al., 2001). В целом некоторые исследователи связывают аутизм с дефицитом глутаматергической системы в мозге (Сarlsson, 2015).

Ранее нами было показано, что в условиях хронического социального конфликта, вызванного повторным опытом побед или поражений в ежедневных межсамцовых конфронтациях (Kudryavtseva, 1991), у самцов мышей формируются не только повышенная тревожность, агрессивность или депрессивноподобное поведение, но и нарушения коммуникативного поведения и социального взаимодействия. Такие животные демонстрировали сниженную коммуникативность, а также стереотипные формы поведения (аутогруминг, вставание на задние лапы, кружение, разрывание и разбрасывание подстилки, повороты в прыжке и др.), что позволяет рассматривать данную модель как модель, воспроизводящую некоторые черты аутистического поведения (Коваленко, Кудрявцева, 2010), формирующиеся под влиянием социальной среды. При этом наши предыдущие исследования показали, что в этот процесс были вовлечены гены аутистического спектра, экспрессия которых изменялась под влиянием агонисти- ческих взаимодействий (Kudryavtseva et al., 2017).

В связи с этим целью настоящей работы было изучить у животных с альтернативным опытом социального пове- дения, позитивным и негативным, взаимосвязь изменения экспрессии генов, кодирующих белки, вовлеченные в функционирование ГГ, и генов, связанных с патологией аутизма (ГА) в гиппокампе. Выбор этой структуры головного мозга основан на многочисленных литературных данных о том, что гиппокамп непосредственно участвует в патогенетических процессах аутизма (DeLong, 1992), в развитии тревожных расстройств (Irle et al., 2010) и депрессии (Savitz, Drevets, 2009). Гиппокамп отвечает за эмоциональную саморегуляцию, обучаемость и память. А, как известно, нарушение именно этих функций часто наблюдается у людей, демонстрирующих симптомы аутистического спектра.

## Материалы и методы

Животные. Эксперименты проводили на самцах мышей линии С57BL/6J в возрасте 2.5 мес. и массой тела 26–28 г. Животные были привезены из питомника лабораторных животных Института биоорганической химии Российской академии наук (Пущино, Московская область). Эксперимент проводили в виварии конвенциональных живот- ных Института цитологии и генетики Сибирского отде- ления РАН. Воду и корм (гранулы) животные получали в достаточном количестве. Световой режим был 12С : 12Т. Все процедуры осуществляли в соответствии с между- народными правилами проведения экспериментов с жи- вотными (Directive 2010/63/EU of the European Parliament and of the Council on the Protection of Animals Used for Scientific Purposes). Применяемые методики для изучения поведения у мышей были одобрены Научной комиссией (№ 9) Института цитологии и генетики СО РАН (Протокол № 613 от 24.03.2010).

Поведенческие исследования. Для формирования альтернативного опыта социального поведения у самцов мышей использовали модель сенсорного контакта (Kudryavtseva et al., 2014). Животных попарно помещали в экспериментальные клетки, разделенные пополам про- зрачной перегородкой с отверстиями, позволявшей мы- шам видеть, слышать, воспринимать запахи друг друга (сенсорный контакт), но предотвращавшей физическое взаимодействие. Ежедневно во второй половине дня (15:00–17:00) убирали перегородку, что приводило к агонистическим взаимодействиям. В течение первых двух-трех дней тестов выявляли победителей (агрессоров/ агрессивных животных) и особей, терпящих поражения (жертв) при взаимодействии с одним и тем же партнером. В дальнейшем ежедневно после теста побежденного самца пересаживали в новую клетку к незнакомому агрессивно- му партнеру, сидящему за перегородкой. Взаимодействие самцов прекращали, если интенсивные атаки со стороны нападающей особи во время агрессивных столкновений длились более трех минут, устанавливая между ними перегородку. В исследование были взяты агрессивные животные с 20-дневным опытом побед (агрессоры) и с 20-дневным опытом поражений (жертвы). В качестве контроля использовали самцов, не имевших последова- тельного опыта агонистических взаимодействий. В каж- дой группе было по 14–16 животных.

Количественную оценку реакции экспериментальных животных на незнакомого партнера на нейтральной территории проводили при помощи теста «социальные взаимодействия » (Kudryavtseva et al., 2017). Как параметры социального поведения мы рассматривали избегание незнакомого партнера или же замирание при его подходе и приближение к партнеру: подходы, обнюхивания, сле- дование за партнером; как параметры индивидуального поведения выделяли стойки – вставания на задние лапы, являющиеся показателем исследовательской активно- сти, аутогруминг, служащий показателем смещенной активности и неадекватного стереотипного поведения, и двигательную активность, оценивающую интенсивность передвижений по клетке.

Статистическую обработку полученных данных вы- полняли с использованием пакета программ STATISTICA (ver. 8.0; StatSoft, Inc., 2001). Проверка нормальности рас- пределения количественных признаков была проведена с использованием критерия Шапиро-Уилка (Shapiro-Wilk’s W-test). Поскольку выборки исследованных параметров поведения удовлетворяли гипотезе о нормальном рас- пределении, были использованы методы параметрической статистики: однофакторный дисперсионный анализ (ANOVA) с фактором «группа» (контроль, агрессивные самцы, жертвы); последующее попарное сравнение по- казателей осуществляли с помощью LSD-теста Фишера.

При помощи корреляционного анализа по методу Пирсона мы исследовали взаимосвязь экспрессии ГГ и ГА. В экспериментальных поведенческих группах было по 10–12 животных.

RNA-Seq-анализ проводили с помощью ЗАО «Гено- аналитика» (http://genoanalytica.ru, Москва). Методика анализа подробно описана ранее (Галямина и др., 2017).

Мы провели проверку результатов, сравнив их с дан- ными B.M. Kadakkuzha (Kadakkuzha et al., 2015), пред- ставившими полный транскриптомный анализ генов в гиппокампе интактных мышей. Обнаружен высокий уровень корреляции (0.74 по Спирмену) между экспрес- сией генов аутизма и ГГ у контрольных особей в нашем эксперименте и у интактных животных в работе (Kaddakuzha et al., 2015), что может быть дополнительным до- казательством адекватности применяемого метода. Кро-ме того, осуществляли кросс-верификацию результатов (Babenko et al., 2017) с данными, полученными в Стэнд- фордском университете (Zhang et al., 2014), и обнаружили значительную корреляцию между ними. Это доказывает, что метод транскриптомного анализа позволяет выявить происходящие в мозге молекулярные процессы.

В качестве верификации данных настоящего экспери- мента использовали результаты, полученные нами ранее при помощи RT-PCR (Smagin et al., 2013), экспрессия ко- торых, по методу RNA-Seq, была изменена. Было показано совпадение направленности и выраженности изменений экспрессии для генов, кодирующих белки серотонерги- ческой системы в ядрах шва среднего мозга, полученных при применении этих методов, что позволяет говорить о высокой надежности результатов этого исследования и о стабильности примененного метода.

Декапитацию всех трех групп экспериментальных животных проводили одновременно на следующий день после последней конфронтации. Гиппокамп извлекался одним исследователем в соответствии с анатомическим атласом мозга (Allen Mouse Brain Atlas; http://mouse.brainmap.org/static/atlas). Все образцы помещали в рас- твор RNAlater ( LifeTechnologies, США) и хранили при температуре –70 °С до секвенирования. Проводили два типа сравнения: контроль–агрессоры и контроль–жертвы.

Для изучения изменений глутаматергической системы в гиппокампе животных с нарушенным социальным по- ведением были исследованы: гены переносчиков глутама- та: Slc17a6, Slc17a7 и Slc17a8; гены, кодирующие 1–4-ю субъединицы ионотропного AMPA-рецептора: Gria1, Gria2, Gria3, Gria4; гены, кодирующие 1–5-ю субъеди- ницы ионотропного каинатного глутаматного рецептора: Grik1, Grik2, Grik3, Grik4, Grik5; гены, кодирующие 1, 2а, 2b, 2c, 2d, 3a, 3b субъединицы ионотропного NMDA- рецептора: Grin1, Grin2a, Grin2b, Grin2c, Grin2d, Grin3a, Grin3b; гены, кодирующие метаботропные рецепторы 1–8-го подтипов: Grm1, Grm2, Grm3, Grm4, Grm5, Grm6, Grm7, Grm8; гены, кодирующие 1 и 2-ю субъединицы глу- таматного ионотропного рецептора дельта Grid1 и Grid2; GRID2IP – белок, взаимодействующий с Grid2; гены фермента глутаматдекарбоксилазы, метаболизирующей глутамат в ГАМК: Gad1 и Gad2.

По генетическим базам данных OMIM (http://omim.org/), GeneCards (http://www.genecards.org/), MalaCards (http://www.malacards.org/) из 1.5 тыс. аннотированных генов было выбрано около 80 основных генов-кандидатов аутизма, которые в дальнейшем были просмотрены в гиппокампе у контрольных особей и животных с нарушенным социальным поведением. При анализе сравнивали по три пробы от каждой группы животных.

## Результаты

Исследование нарушений социального поведения у самцов мышей под влиянием хронического социального стресса. ANOVA выявил достоверное влияние фактора «группа» (контроль, агрессоры, жертвы) на избегание партнера (F(2.29) = 52.30, p < 0.001), приближение (F(2.28) = 1097.4, p < 0.001), время стоек (F(2.29) = 661.7, p < 0.001), время двигательной активности (F(2.29) = 2549, p < 0.001). Сравнение групп LSD-тестом Фишера (рис. 1) выявило увеличение времени избегания у жертв, по срав- нению с контролем и агрессивными животными ( p < 0.001 для обеих групп). По сравнению с контролем у агрессоров и жертв также было показано снижение времени стоек ( p < 0.001 для обеих групп) и времени приближения к партнеру ( p < 0.001 для обеих групп). Кроме того, у жертв время приближения к партнеру было снижено и по сравнению с агрессивными животными ( p < 0.006). Выявлены увеличение времени двигательной активности у агрессоров ( р < 0.001) и снижение времени у жертв ( р < 0.042), по сравнению с контрольными животными. Время двигательной активности было значительно ниже у жертв, по сравнению с агрессорами ( р < 0.001). Кроме того, у жертв были увеличены число и время аутогруминга, по сравнению с контролем ( p < 0.041, p < 0.034 соответственно).

**Fig. 1.**
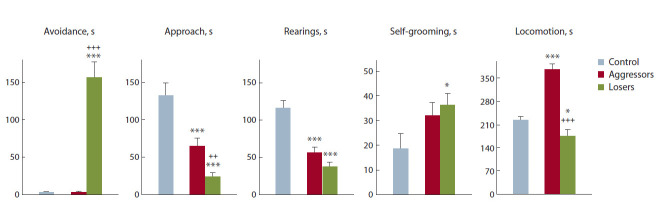
Behavior of the aggressors and losers in the social interactions test. * p < 0.05; *** p < 0.001 vs control; ++ p < 0.01; +++ p < 0.001 – losers vs aggressors.

Таким образом, мы видим, что в тесте «социальные взаимодействия» жертвы активно избегали незнакомого партнера (тестера) на нейтральной территории. Они редко первыми подходили и проявляли интерес к незнакомому партнеру, в отличие от контроля. Контрольные животные целенаправленно следовали за партнером, обнюхивали его. У жертв также было снижено время стоек, рассматрассматриваемое нами как показатель исследовательской актив- ности. Кроме того, у них было увеличено время демон- страции аутогруминга, который может оцениваться как признак стереотипного поведения. При этом отмечено, что у агрессивных животных были снижены параметры коммуникативности, что может свидетельствовать о на- рушениях социального поведения. Большую часть 10-ми- нутного теста (около 5–6 мин) агрессоры хаотично пере- мещались по клетке, не обращая внимания на партнера: общее время двигательной активности было значительно больше, чем у жертв, что может отражать развитие гиперактивности и, по-видимому, дефицит внимания. Ранее нами было показано, что в агонистических взаимодей- ствиях агрессоры часто демонстрируют быстрые пово- роты в прыжке или же повторяющиеся повороты вокруг оси тела в тесте «перегородка» (Kudryavtseva, 2006), т. е. стереотипии у агрессивных мышей этой линии могут проявляться в другом тесте и другой форме. Таким об- разом, и у агрессоров, и у жертв в результате 20-дневных агонистических взаимодействий развиваются симптомы аутистического спектра.

Исследование экспрессии ГГ и ГА у самцов мышей с контрастными типами социального поведения. В результате анализа данных RNA-Seq в гиппокампе обна- ружено изменение экспрессии девяти генов-кандидатов аутизма (рис. 2, табл. 1). Так, у жертв выявлено увеличение экспрессии генов Shank2 ( p < 0.040), Nlgn2 ( p < 0.047), Pcdh10 ( p < 0.011), Reln ( p < 0.026) и Arx ( p < 0.0002), по сравнению с уровнем экспрессии у контрольных животных. У агрессивных мышей в гиппокампе под влиянием повторного опыта агрессии в межсамцовых конфрон-тациях обнаружено снижение экспрессии генов Shank3 ( p < 0.010), Auts2 ( p < 0.023), Ctnnd2 ( p < 0.020), Nrxn2 ( p < 0.010).

**Fig. 2.**
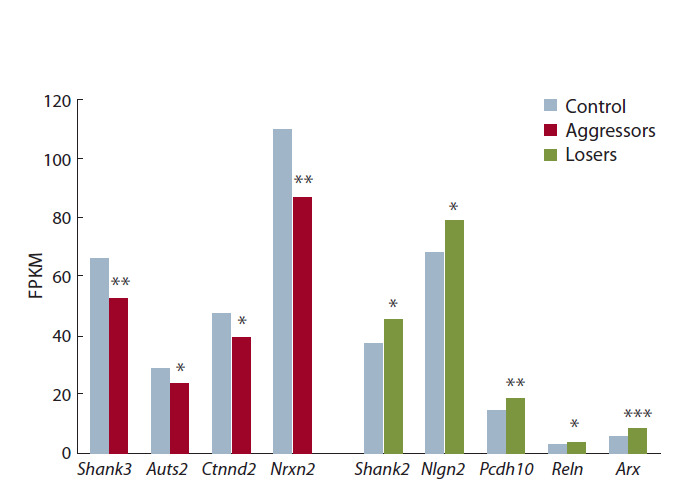
Change in GA expression in the hippocampus of aggressors and losers. * р < 0.05; ** p < 0.01; *** p < 0.001 – vs control.

**Table 1.**
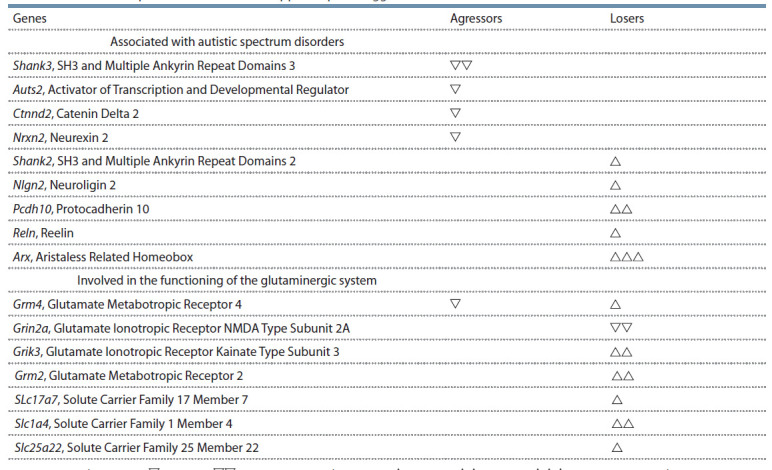
Differential expression GG and GA in hippocampus of aggressors and losers Note: Decreased expression: – p < 0.05; – p < 0.01. Increased expression: – p < 0.05; – p < 0.01; – p < 0.001 vs control.

Были проанализированы изменения уровня экспрессии генов, кодирующих белки, вовлеченные в функциони- рование ГГ в гиппокампе мышей (см. табл. 1, рис. 3). Показано, что у мышей в гиппокампе под влиянием по- вторного опыта агрессии в межсамцовых конфронтациях снижена экспрессия гена Grm4 ( p < 0.023), кодирующего метаботропный рецептор 4-го подтипа. У жертв было обнаружено снижение экспрессии гена Grin2a ( p < 0.01), кодирующего субъединицу 2а ионотропного NMDA-рецептора, при этом повышалась экспрессия генов Grm2 ( p < 0.004) и Grm4 ( p < 0.02), кодирующих метаботроп- ные рецепторы 2 и 4-го подтипов, гена Grik3 ( p < 0.003), кодирующего субъединицу 3 ионотропного каинатного глутаматного рецептора, генов переносчиков глутамата Slc17a7 ( p < 0.051), Slc1a4 ( p < 0.01), Slc25a22 ( p < 0.028).

**Fig. 3.**
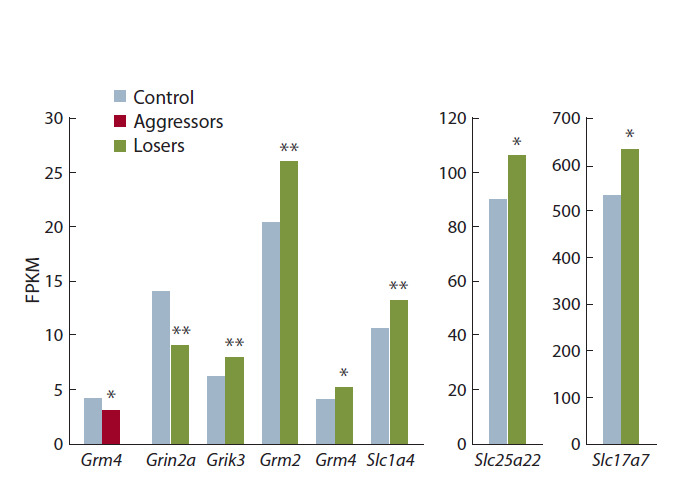
Change in expression of GG in the hippocampus in mice with a disturbances of social behavior. * р < 0.05, ** p < 0.01 – vs control.

Результаты корреляционного анализа продемонстри- ровали высокий уровень корреляционной взаимосвязи между экспрессией ГГ и ГА у животных исследуемых групп (см. табл. 1 и 2). Корреляция между уровнями экс- прессии ГА и ГГ показывает возможное участие глута- матэргической системы в механизмах этого заболевания. Из ГА наибольшее число корреляций обнаружено для генов Nlgn2, Pcdh10, Arx, Ctnnd2, Nrxn2. В то же время уровень экспрессии гена Reln коррелирует только с уров- нем экспрессии гена Slc1a4.

**Table 2.**
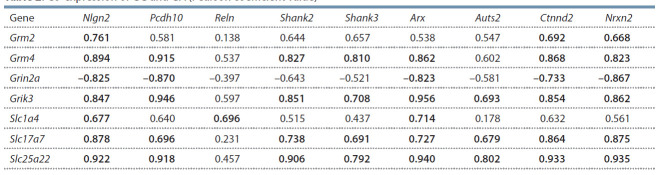
Co-expression of GG and GA (Pearson coefficient value)

Значения экспрессии некоторых ГГ и ГА в единицах FPKM, между которыми установлена статистически значимая коррелятивная взаимосвязь, показаны на рис. 4. Большая часть генов демонстрирует положительную корреляцию друг с другом. Например, экспрессия Grm4, единственного гена, который изменил экспрессию и у жертв, и у агрессоров, положительно коррелирует с экспрессией ГА: Shank3 (r = 0.810, p < 0.01) и Arx (r = 0.862, p < 0.01) (см. рис. 4, а). Экспрессия гена Grin2a демонстрирует отрицательную корреляцию с экспрессией ГА, например генов Nlgn2 (r = –0.823, p < 0.01) и Arx (r = –0.823, p < 0.01) (см. рис. 4, б ). Единственный ген, с которым коррелирует уровень экспрессии гена Reln, – это Slc1a4 ( r = 0.696, p < 0.05) (см. рис. 4, в).

**Fig. 4.**
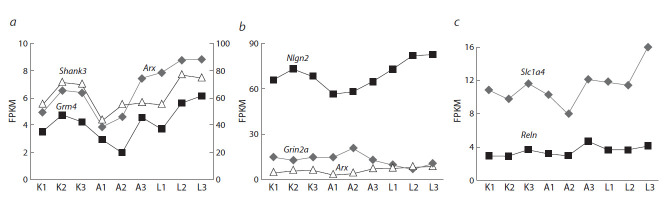
Correlating GG and GA. K1–3 – control animals; A1–3 – aggressors; L1–3 – losers. a, The right axis of ordinates is for the Shank3 gene, the left one is Arx and Grm4.

## Обсуждение

В наших предыдущих работах было обнаружено, что после 20 дней агонистических взаимодействий и проживания в условиях хронического социального конфликта у экс- периментальных самцов нарушаются многие параметры социального поведения (Коваленко, Кудрявцева, 2010), схожие по симптоматике с заболеваниями аутистического спектра. Мы считаем, что в данном случае коморбидные аутистические симптомы развиваются на фоне других заболеваний. Это может быть тревожно-депрессивное состояние, возникающее вследствие хронического социального стресса у самцов мышей С57Bl/6 (Berton et al., 2006; Kudryavtseva et al., 2006) или каталепсии, развивающейся у животных СВА/Lac ( Kudryavtseva et al., 2006), или же патологической агрессии (Kudryavtseva, 2006), формирующейся под влиянием повторного опыта агрессии. В литературе существуют данные, свидетельствующие, что коморбидность аутистических симптомов и расстройств настроения может быть следствием работы одних и тех же генов, продукты которых вовлекаются в патофизиологические механизмы развития этих заболеваний (Ragunath et al., 2011).

В связи с этим помимо генов, для которых показано участие в развитии симптомов аутизма, мы исследовали также изменения экспрессии ГГ (транспортеров, рецепторов, ферментов катаболизма), так как известно, что эта нейромедиаторная система вовлечена в развитие данной патологии (Сarlsson, 2015).

Мы выявили в гиппокампе изменение экспрессии семи ГГ: ген Grin2a кодирует NR2А-субъединицу NMDA- рецептора; Grm2 и Grm4 – метаботропные глутаматные рецепторы, mGluR2 и mGluR4 соответственно; Grik3 – субъединицу каинатного рецептора, Slc1a4 и Slc17a7 – транспортеры глутамата Vglut1; Slc25a22 – белок митохондриального переносчика глутамата. Изменили свою экспрессию 9 генов, ассоциированных с заболеваниями аутистического спектра: Shank2, Shank3, Nlgn2, Reln, Arx, Nrxn2, Auts2, Ctnnd2, Pcdh10.

Белки семейства SHANK, известные также как ProSAP, являются строительными белками на возбуждающих глутаматергических синапсах. Так, ген Shank2 кодирует белок, взаимодействующий на постсинаптической мембране с NMDA-рецептором, участвуя в синаптической глутаматергической передаче (Сarlsson, 2015). Вовлечение в процесс развития аутизма генов Shank было впервые описано для Shank3. Например, было отмечено, что мыши, гетерозиготные по белку Shank3 (Shank3+/delta-C), демонстрировали низкий уровень социального распознавания и коммуникативности (Wang et al., 2011). В литературе было показано, что для аутистических симптомов характерно снижение функции Shank2 (Won et al., 2012). Нарушения в социальном поведении характерны как для пониженного (Peça et al., 2011), так и для повышенного (Moessner et al., 2007) уровня белка SHANK3. В нашей работе также об- наружены повышенный уровень экспрессии гена Shank2 и сниженный уровень гена Shank3. Вероятно, нарушения в социальном поведении могут быть связаны с любыми изменениями в функционировании этих белков. Известно, что ProSAP2/Shank3 влияет на работу глутаматергических синапсов, взаимодействуя с внеклеточным С-концом нейролигинов (Meyer et al., 2004). Мутации в этом гене ухудшают синаптическую передачу (Arons et al., 2012). Следовательно, можно предположить, что влияние белков SHANK на развитие аутистических симптомов осуществляется через изменение функционирования глутаматер- гической системы.

Значительную часть ГА, изменивших свой уровень экспрессии в нашей работе, составляют гены клеточной адгезии: Nlgn2 – нейролигин 2-го типа; Pcdh10 – протокадерин; Ctnnd2 – δ-катенин; Nrxn3 – нейрексин 3. Для всех этих белков многократными исследованиями было показано участие в формировании социального поведения и расстройств аутистического спектра. Известно, что изменение уровня белка NLGN2 может влиять на социальное и эмоциональное поведение (Maćkowiak et al., 2014). У аутистических больных обнаружены мутации в генах Pcdh10 (Anitha et al., 2013), Ctnnd2 (Turner et al., 2015), Nrxn3 (Vaags et al., 2012). Кроме того, продукты этих генов взаимодействуют с глутаматергической системой. Так, например, нейролигины вызывают кластеризацию Vglut- положительных синаптических пузырьков (Graf et al., 2004), без чего невозможно созревание и функционирова- ние синапса. Правда, там же отмечено, что это характерно для нейролигина 1, а нейролигин 2 не располагается на одном синапсе с транспортером глутамата. Обнаружено, что в отсутствие нейролигинов снижается число Vglut1- положительных терминалей (Chih et al., 2005). Можно предположить, что на самом деле нейролигин взаимодействует непосредственно с NMDA-рецепторами, а переносчик Vglut1 выступает только в качестве маркера этих ре- цепторов. Что касается продукта гена Pcdh10, то установлено, что протокадерины локализуются на нейронах, фор- мирующих глутаматергические AMPA и каинатные рецепторы (Puller, Haverkamp, 2011). Кроме того, показано, что у мышей, нокаутных по генам протокадеринов, снижено число глутаматных транспортеров Vglut 1–2 (Chen et al., 2012), что также свидетельствует о взаимосвязи между протокадеринами и глутаматергической системой.

Продукты генов Arx и Reln (белок рилин) играют важную роль в механизмах пре- и постнатального нейрогенеза. Участие этих генов в патофизиологии аутизма является спорным: часть авторов приводят данные в пользу этого факта (Wall et al., 2009), в то время как другие опровергают его (Persico et al., 2001). В то же время обнаружена связь этих генов с функционированием глутаматергической системы. Так, известно, что мутации в гене Arx связаны с изменениями в глутаматергической системе у больных эпилепсией (Beguin et al., 2013), а белок рилин способен повышать мобильность рецепторов, содержащих NR2_B_-субъединицу, снижая время ее пребывания в синапсе, таким образом, изменяя состав рецепторов в сторону преобладания NR2_А_-субъединицы (Groc et al., 2007).

Ген Auts2 кодирует экспрессирующийся в головном мозге белок с неизвестной функцией, хотя предполагают, что он вовлечен в механизмы развития нервной системы (Oksenberg et al., 2013). Мутации в этом гене обнаружены у пациентов с аутистическими расстройствами (Liu et al., 2015). Показано, что Auts2 экспрессируется на глутаматергических нейронах гиппокампа (Hori et al., 2014).

Так как большинство ГА, изменивших экспрессию в нашем исследовании, – гены, связанные с клеточным скелетом и внеклеточным матриксом, в частности участвующие в формировании синапсов, а ГГ – гены, кодирующие субъединицы рецепторов, то можно полагать, что изменение активности глутаматергической системы проявляется в аутистических симптомах.

Корреляционный анализ наших экспериментальных данных в целом выявил высокий уровень корреляции между экспрессией ГГ и ГА. Исключение составил ген Reln, коррелирующий только с геном Slc1a4. Характерно также, что везде обнаруживается положительная корреляция между генами, в то время как ген Grin2a отрицательно коррелирует с ГА. Однако снижение экспрессии Grin2a, продукт которого является субъединицей NMDA- рецептора, хорошо согласуется с литературными данными, связывающими развитие аутистических симптомов с дефицитом этих рецепторов (Lee et al., 2015). Экспрессия гена Nlgn2 коррелирует с экспрессией всех ГГ, экспрес- сия генов Pcdh10, Arx, Cttnd2, Nrxn2 – с экспрессией практически всех генов. Это позволяет предположить, что именно при помощи данных генов ГГ вовлекается в механизмы развития аутистического поведения. Можно видеть, что все наиболее коррелирующие гены, кроме Arx, – гены клеточной адгезии. Мы предполагаем, что продукты этих генов участвуют в соединении синапсов, в том числе глутаматергических, с клеточной мембраной. Таким образом, они вовлекаются в функционирование глутаматергической системы. Следовательно, изменение экспрессии этих генов изменяет уровень активности ГГ, что, в свою очередь, приводит к изменениям в социальном поведении, хотя нельзя исключить и обратный вариант взаимодействия.

## Заключение

Проведенное исследование подтвердило полученные ранее результаты, свидетельствующие о том, что под влиянием хронического социального конфликта у сам- цов мышей могут развиваться нарушения социального поведения, причем в этом процессе участвуют как гены, кодирующие белки, вовлеченные в функционирование глутаматергической системы, так и гены, связанные с патологией аутизма. Коэкспрессия этих генов позволяет говорить о вовлечении глутаматергической системы головного мозга в развитие патологии социального по- ведения. Полученные результаты могут служить дока- зательством того, что аутистические симптомы могут не только быть следствием генетических нарушений, но и развиваться в течение жизни индивидуума под влиянием стрессорных воздействий.

## Conflict of interest

The authors declare no conflict of interest.
